# Prevalence of Psychological Distress and Its Correlation With Sudanese Medical Students’ Sociodemographic Characteristics: A Cross-Sectional Study 2022

**DOI:** 10.1192/bjo.2025.10142

**Published:** 2025-06-20

**Authors:** Khalid Yeddi, Tawheed Abdelfatah Ahmed, Danya Ibrahim, Kamil M. A Shaaban

**Affiliations:** ^1^Khartoum University, Faculty of Medicine, Khartoum, Suda; ^2^Department of Psychiatry, Khartoum University, Faculty of Medicine, Khartoum, Sudan

## Abstract

**Aims:** To determine the prevalence of psychological distress among Sudanese medical students and its association with their sociodemographic characteristics.

**Methods:** In this cross-sectional study, 353 Sudanese medical students completed an online questionnaire containing socio-demographic data such as gender, age, year of study, marital status, monthly income, and residency. Additionally, the Depression, Anxiety, and Stress Scale-21 (DASS-21) items were employed to gauge the levels of psychological distress among the participants and to explore the association with the demographic data. Utilizing the Pearson chi-square test, the analysis delved into the associations between socio-demographics data and psychological distress.

**Results:** Anxiety was the most prevalent psychological distress among medical students, as 76.8% of them exhibited anxiety symptoms. This was followed by depression, with a prevalence of 70.2%, and 56.7% of the students reported suffering from stress. The study found significant associations between depression and stress with age and gender, as well as a significant association of anxiety with age.

**Conclusion:** A considerable number of medical students are experiencing psychological distress. It is recommended to implement intervention programmes to educate Sudanese medical students about mental health issues and psychological distress.

